# RUNNING AND WALKING IN DEMENTIA AND MILD COGNITIVE IMPAIRMENT: A SCOPING REVIEW

**DOI:** 10.1093/geroni/igad104.2555

**Published:** 2023-12-21

**Authors:** Benjamin Mast, Fairouz Gaballa, Dylan Gillespie, Kaitlyn Graves, Darby Simon, Emilee Ertle, Nina Sanfilippo, Romina Babazadeh

**Affiliations:** University of Louisville, Louisville, Kentucky, United States; University of Prince Edward Island, Charlottetown, Prince Edward Island, Canada; University of Prince Edward Island, Charlottetown, Prince Edward Island, Canada; University of Prince Edward Island, Charlottetown, Prince Edward Island, Canada; University of Louisville, Louisville, Kentucky, United States; University of Louisville, Louisville, Kentucky, United States; William James College, Newton, Massachusetts, United States; University of Prince Edward Island, Charlottetown, Prince Edward Island, Canada

## Abstract

Growing evidence suggests physical exercise may reduce dementia risk, but there has been little investigation of specific forms of exercise for people living with dementia and cognitive impairment. Prior reviews show running and walking have mental health benefits in the general adult population, but no reviews of walking and running among people living with dementia and mild cognitive impairment have been published. A scoping review was conducted to identify and evaluate evidence concerning whether running and walking affect dementia risk, improve/maintain cognitive functioning, and improve quality of life and psychological well-being. PsycInfo, Medline, CINAHL, and SPORTDiscus were searched to identify studies with the target population (people living with dementia or mild cognitive impairment) and target activities (running, jogging, walking). 1153 records were uploaded into Covidence and 821 studies were excluded based on abstract reviews. Full text review yielded 49 papers. Forty-seven studies examined walking and a broad range of outcomes: cognition, ADL functioning, quality of life, behavioral expressions of distress, social participation, and identity. The remaining two studies examined (1) a combination of running and walking among people with mild cognitive impairment and (2) the link between cognitive decline and a history of running, jogging, and walking (past ten years). This review shows growing interest in walking as an intervention with many possible benefits for people living with dementia or mild cognitive impairment. Studies of jogging and running are still relatively rare in this population. Additional contributing authors: Hannah Gardner, Clarissa Geibel, Katherine King, Jessica Strong, Travis Saunders, Christine Wise.

